# Study on blasting fragmentation mechanism of burnt rock in open-pit coal mine

**DOI:** 10.1038/s41598-024-55802-2

**Published:** 2024-02-29

**Authors:** Zheng-Zhao Jia, Hong-Jie Li, Wei Li, Jie Yan, Xiao-He Wang

**Affiliations:** 1 China Coal Research Institute, Beijing, 100013 China; 2 School of Energy and Mining Engineering, China University of Mining and Technology (Beijing), Beijing, 100083 China

**Keywords:** Open-pit mine, Burnt rocks, Bench blasting, Blasting mechanism, Coal, Natural hazards

## Abstract

A large number of burnt rocks in some open-pit mines in Xinjiang, Inner Mongolia and Ningxia have a great influence on the blasting effect. For this kind of rock, through the analysis of physical and chemical changes, combined with ANSYS/LS-DYNA and PFC 2D numerical simulation software, a burnt rock model with multiple joint cracks and irregular distribution is constructed to simulate the blasting process of burnt rock under the combined action of stress wave and detonation gas. The results show that the fracture of rock mass affects the propagation of blasting cracks in the fracture area, resulting in stress concentration and stress hindrance. The action time of stress wave is reduced, and the energy of blasting gas is partially absorbed by the fracture, resulting in uneven stress on the burnt rock bench and seriously affecting the bench blasting effect.

## Introduction

With the rapid development of open-pit coal mines, the drilling and blasting technology, which has an important impact on open-pit mining, has also been greatly improved with the progress of science and technology. The development of blasting technology aims at refinement, greening and intelligent automation, and continuous innovation and improvement.Since 1613, the development of blasting theory has gone through the initial stage, the establishment stage of blasting rock mechanics theory and the development stage of rock damage and fracture theory^1–3^. In the first two stages, the research is mostly based on conventional and homogeneous sandstone and conglomerate. However, in the actual blasting engineering, there are often a large number of weak structural planes such as joints and faults in the rock mass, which makes the blasting theory studied under ideal conditions (Homogeneous rock). There is a deviation in the actual blasting application, which increases the difficulty of blasting technology in the application of open pit mines.In the latest development stage of blasting theory research, many scholars have studied the blasting mechanism of fractured media from the internal structure characteristics of rock mass, and produced some new viewpoints on blasting rock breaking theory. Rock mass elastoplastic theory, fracture theory, damage theory and so on came into being^4–8^. Many scholars have analyzed the causes and development rules of macroscopic cracks in rock mass by means of theoretical analysis and laboratory experiments, and summarized the influence of structural plane on stress wave propagation in fractured rock mass. Xie H P et al. proposed a fractal model of crack propagation by combining rock crack bifurcation and fractal geometry. They believed that the sub-cracks derived from the rock were extended on the basis of the previous group of cracks, and each group of cracks had self-similarity. Based on this research, the model was used to determine the relationship between stress and fractal dimension^9,10^. Fordyce et al.^11^ pointed out that when the width of the joint fracture in the rock mass is smaller, the wave impedance of the internal filling is closer to that of the surrounding rock, and the absorption of strain energy is less. Yang et al. based on the rock crushing mechanism and damage mechanics described the process of rock damage and fracture under blasting as two stages: the initial stage of fracture under the action of explosive stress wave and the later stage of quasi-static action of explosive gas, and determined the damage field of rock under explosive load through theoretical derivation formula^12^. Wang combined fracture mechanics with damage mechanics in the article, systematically expounded the damage and fracture mechanism of rock, and established the whole process model of rock damage under blasting action^13,14^. Zhang^15^ summarized the influence law of blasting effect under different types of joint fissures by establishing different types of joint fissure models. Min et al.^16^, analyzed the influence of structural plane on the propagation of stress wave and the failure of rock mass, and improved the blasting technical measures. Peng^17^ carried out the blasting test of rock mass model with joint fissure in the laboratory, tested the stress wave in each joint of the model, and summarized the propagation law of explosion stress wave in joint fissure rock. Li et al.^18^ obtained the attenuation law of stress wave and energy in layered jointed rock mass by comparing the particle velocity of stress wave in the explosion process of intact rock mass and fractured rock mass model by combining laboratory experiment and numerical simulation. With the help of ANSYS/LS-DYNA software, by adding different widths, materials and angles of joint fissures to the numerical model of homogeneous rock, the propagation law of stress wave and its influence on reducing blasting vibration are summarized^19^. Liu et al. in the process of studying the physical and mechanical properties of rock and the mechanism of blasting fracture, obtained the hypothesis of the combined action of detonation gas and stress wave. In addition, through the comparison of algorithms in ANSYS/LS-DYNA numerical simulation software, it is determined that the ALE algorithm in the software is more suitable for dealing with fluid–solid coupling problems^20^.

**Figure 1:**
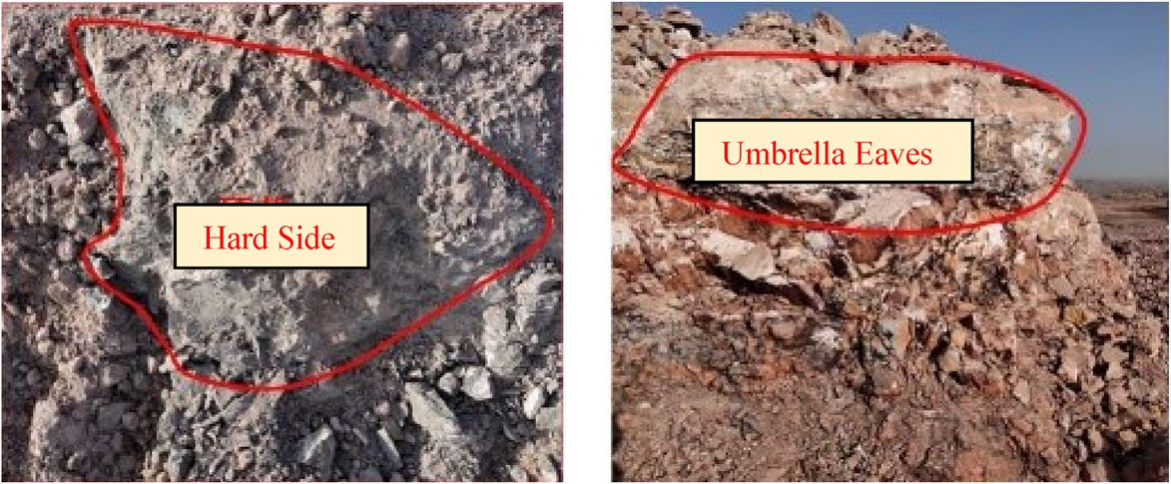
"Hard Side" and "Umbrella Eaves" of working face.

Limited by the scientific and technological conditions and blasting safety requirements during the study, the research methods are limited to laboratory research, and the research objects and models are mostly based on layered joints, resulting in insufficient systematicness and scale in the research process. The same blasting technology has different blasting effects under different rocks.Through on-site observation of the blasting situation in the burnt rock area, the author found that the large block rate after blasting in the burnt rock area was as high as 30%, and accompanied by a large area of 'hard side' and 'umbrella eaves' phenomenon, as shown in Fig. 1 below. The 'hard side', 'umbrella eave' and 'large block' need to be broken mechanically. There are hidden dangers in the operation of mutual operation equipment, and the operation efficiency of excavator and subsequent stripping is reduced.

In the final analysis, the special properties of burnt rock determine the 'good or bad' of blasting effect. Because the burnt rock contains a large number of discontinuities such as fissures and joints with different scales and directions, the rock mass has typical discontinuity and anisotropy. The explosion stress wave and the energy released by the explosive are affected by the cracks and cannot be fully utilized, resulting in unsatisfactory blasting effect or even failure to complete the blasting operation.Therefore, this paper takes burnt rock as the research object. Based on the original blasting theory, the blasting mechanism of rock mass with random joints is studied by means of theoretical analysis, laboratory experiment and numerical simulation, so as to provide guidance for open-pit mine engineering blasting.

## Study on rock characteristics of burnt rock

### Study on the characteristics of burnt rock-rock

#### Test of mechanical properties of burnt rock

The rock is obviously affected by temperature. According to the distance from the spontaneous combustion coal seam, the burnt rock mass can be divided into burnt lava zone, sintered rock zone and baking rock zone. Based on the analysis of the reasons affecting the blasting effect and the needs of numerical simulation, it is necessary to determine the physical and mechanical properties of burnt rock in the laboratory.Based on the engineering rock mass test method 'GBT50266-2013', the uniaxial compressive strength test, tensile strength test (Brazilian splitting method), shear strength test (variable angle method) and rock density test (pycnometer method) were carried out. The test results are shown in Table 1 below.

**Table 1:** Summary of rock property tests in burning area.

Rock type	Compressive strength (MPa)	Tensile strengt (MPa)	Cohesion (MPa)	Angle of internal friction (°)	Elastic modulus (GPa)	Poisson ratio	Density (kg·m^−3^)	Porosity (%)
Burnt lava	48.78	7.28	56.83	15.11	5.96	0.25	2160	6.9
Sintered rock	58.69	5.69	57.48	18.26	5.10	0.30	2030	6.4
baked rock	46.31	6.72	31.89	16.72	10.27	0.18	2290	6.2

#### Test and analysis of mineral composition of burnt rock

**Figure 2:**
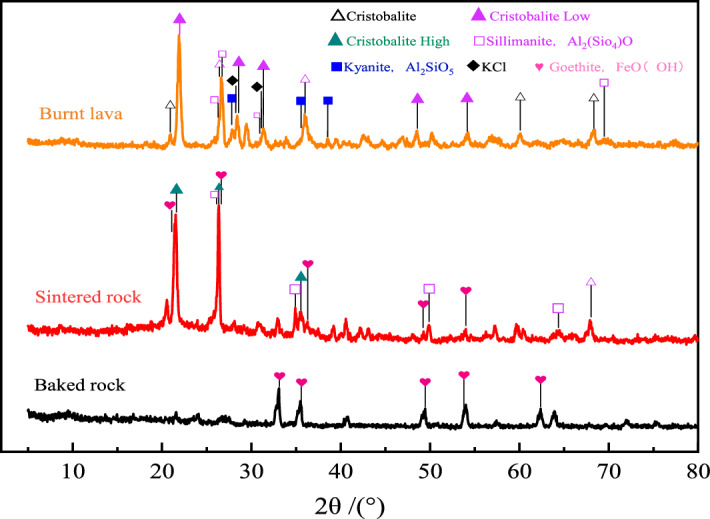
XRD patterns of three rock samples in the burning area.

In order to analyze the actual mineral composition of the three rocks after heating, the burnt rock samples were tested by XRD, and the mineral components contained in the three rocks were obtained. The analysis results are shown in Fig. 2. It can be seen from the figure that both the sintered rock and the baking rock contain SiO_2_ of quartz phase. There are many mineral components in the baking rock, most of which exist in the form of low temperature cristobalite, while the sintered rock contains a large amount of high temperature cristobalite and goethite.Both the sintered rock and the baked rock contain a certain amount of Al_2_SiO_5_ components, mainly in the form of sillimanite (Al_2_ (Sio_4_) O), but in the baked rock, some Al_2_O_3_ exists in the form of kyanite (Al_2_SiO_5_).At the same time, the baking rock also contains a small amount of KCl component. The reason for the different mineral composition is that the sintered rock is closer to the burning coal seam, the baking is more serious, and the product evolved after high temperature oxidation; KCI is easily decomposed by high temperature, so only KCL components are detected in the surface baking rock.Goethite exists in both sintered rock and burnt lava. The content of goethite in sintered rock is low and the mineral composition in burnt lava is dominated by goethite. There is no obvious peak of other components in the burnt lava map, because the existing area directly contacts the spontaneous combustion coal seam, and the formation temperature is the highest. SiO_2_ and Al_2_O_3_ are transformed into amorphous forms, so obvious crystal diffraction peaks cannot be observed.

From the perspective of mineral composition, the strength of burnt rock decreases and is easy to break after high temperature, which can be considered as:

1. The expansion and destruction of the original micro-cracks are aggravated during the precipitation process of the product after the chemical change of the organic matter.
2. The rock is squeezed by thermal stress and internal mineral crystal expansion, resulting in cracks or even broken.

#### Wave impedance Z of burnt rock

In the previous test of the physical and mechanical parameters of burnt rock, the longitudinal wave velocity $C_{p}$ and wave impedance *Z* of the rock were not tested, so the wave impedance *Z* of the burnt rock was obtained by the following formula.

$$C_{p} = \left[ {E\left( {1 - \upsilon } \right)/\rho \left( {1{ + }\upsilon } \right)\left( {1 - 2\upsilon } \right)} \right]^{{{1 \mathord{\left/ {\vphantom {1 2}} \right. \kern-0pt} 2}}}$$

$$Z{ = }\mathop C\nolimits_{{\text{p}}} \times \rho$$

where $E$—Elastic modulus, Pa; $\upsilon$—Poisson ratio; $\rho$—Rock density, kg/m^3^.

The related parameters of the blastability of burnt rock are shown in Table 2 below.

**Table 2:** Burnt rock wave impedance.

	Baked rock	Sintered rock	Burnt lava
Wave impedance (10^6^ km m^−2^ s^−1^)	9.06	7.67	7.97

According to the calculation results, the burnt rock belongs to the medium wave impedance rock [(5–10) × 10^6^ Pa s/m], and its failure is the result of the combined action of stress wave and explosion gas expansion pressure^1^.

### Burnt rock-analysis of factors affecting blasting effect

#### The influence of burnt rock properties on blasting effect

1. Mineral composition affects rock strength.

Because the rock is affected by factors such as high temperature and high pressure for a long time, even the mineral composition of the same rock is different. The mineral composition of sandstone changes after high temperature fire, which directly reduces the strength of rock. In addition, the oxidation reaction and expansion of rock after ablation, baking and stress extrusion lead to the fragmentation of rock mass structure. When it is damaged by explosion, its resistance will also decrease.

2. The influence of burnt rock properties on blasting effect.

Rock strength: In practical engineering, there are often some problems of high blasting difficulty of weak rock (low compressive strength). The reason is that there are differences in the crystallization of mineral particles and the degree of structural density in different rocks. In the actual blasting, the essence of hard rock is more difficult to explode than soft rock is that its tensile strength is much higher than that of soft rock, which also shows that the strength of burnt rock is low, but the blasting effect is poor.

Rock density and porosity: The propagation of explosion stress wave is seriously affected by rock density and porosity. With the increase of rock porosity and the number of joint cracks, the propagation velocity of stress wave in rock decreases accordingly. The burnt rock seriously affects the propagation speed of the stress wave during the blasting process and increases the consumption of the explosive energy propagation process due to the disorderly joints and cracks in the rock mass, the development of holes, and more cracks.

#### The influence of burnt rock mass characteristics on blasting effect

Similar to burnt rock, which is rich in joints and fissures, the occurrence, shape, extension scale, density, spatial distribution geometric characteristics, cementation and filling of structural planes will have a direct impact on the blasting process and the final blasting effect. The influence of the structural plane on the blasting effect has six effects, such as stress concentration, stress wave reflection enhancement, energy absorption, energy release, wedge, and changing the direction of blasting action. It can be seen that the burnt rock mass has many types of structural planes, large differences and high degree of fragmentation. The diversity of rock types and the uneven strength of rock are the main reasons that affect the final blasting effect.

#### The influence of structure on stress wave propagation

The stress wave will be reflected and refracted many times in the granular structure rock mass, which will change the direction of stress wave propagation, reduce the propagation speed and accelerate the attenuation speed. Therefore, this kind of rock mass has the greatest influence on the propagation of stress wave, which will seriously affect the blasting effect.

## Combined method to establish blasting numerical model

In this section, based on the type of blasting failure of burnt rock, the combination of LS-DYNA/PFC two numerical simulation methods is used to simulate the joint failure of stress wave and explosion gas expansion pressure. The reliability of the numerical simulation method is verified by analyzing the mechanism of conventional rock blasting.

### Numerical simulation parameter calibration settings

#### Macro parameters of rock

In the previous paper, the physical and mechanical parameters of burnt rock have been measured in the laboratory. It is known that three kinds of rocks in the rock layer are mixed with each other. In the numerical simulation, the physical and mechanical parameters of the sintered rock with the most content in the rock layer are selected as the macroscopic parameters of the material, as shown in Table 3 below.

**Table 3:** Macro mechanical parameters of numerical simulation.

Compressive strength (MPa)	Tensile strength (MPa)	Cohesion (MPa)	Angle of internal friction (°)	Elastic modulus (GPa)	Poisson ratio	Density (kg·m^−3^)
58.69	5.69	57.48	18.26	5.10	0.30	2030

#### Microscopic parameters of rock

In PFC numerical simulation, the mechanical behavior between particles defined in the parallel bond model needs to be set with microscopic parameters. At present, there is no way to obtain microscopic parameters directly through real experiments. It is generally believed that if the mechanical behavior of rock in numerical simulation experiments is similar to that in real physical experiments, the selected microscopic parameters are considered reasonable.

**Figure 3:**
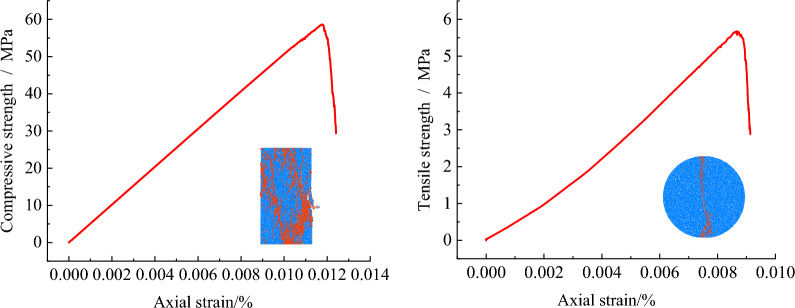
Model microscopic parameter calibration simulation process.

According to the mechanical parameters in Table 3, the numerical simulation parameters of uniaxial compression test (left) and Brazilian splitting test (right) are inverted in PFC as shown in Fig. 3 above. By adjusting the microscopic parameters of particles until the numerical simulation results match the experimental results, the microscopic parameters of particles in the numerical simulation model are finally determined, as shown in Table 4.

**Table 4:** Microscopic parameters of burn rock model.

Microscopic parameters	Minimum radius of particles (mm)	Particle radius ratio	Particle density (kg·m^−3^)	Elastic modulus (GPa)
Values	2.5	1.66	2160	3.45
Poisson ratio	Contact modulus (GPa)	Force of cohesion (MPa)	Friction factor	Angle of internal friction (°)
0.27	2.95	7	0.5	32

### Combined method to obtain blasting load

LS-DYNA nonlinear finite element method can accurately simulate the stress wave generated by explosive explosion through its built-in explosive equation, but it is difficult to simulate the fracture of rock mass during blasting. PFC discrete element method can effectively simulate the fracture and throwing of rock mass, but only through the empirical formula, the dynamic load is simplified as the blasting triangular wave, which is used to describe the stress field of the explosive elastic zone. This method is different from the actual blasting dynamic load time history curve, and does not consider the effect of blasting gas. Therefore, in order to simulate the blasting process of rock mass, considering the combined effect of explosive stress wave and explosive gas, LS-DYNA is used to simulate the stress wave of explosive explosion, Weibull distribution function is used to simulate the effect of explosive gas, and PFC is used to simulate the rock mass medium, so as to realize the generation of cracks in the blasting process of rock mass.

#### Explosion shock wave loading

In this paper, ANSYS/LS-DYNA finite element software is used to simulate the explosive explosion process, and the SHELL element axisymmetric algorithm formula is used to realize the rock blasting process. Through the numerical model of explosive in LS-DYNA software, the explosive type is freely selected, the explosive parameters are set, and the real value of blasting dynamic load is calculated relatively accurately.

The *MAT_HIGH_EXPLOSIVE_BURN material model is selected to describe the explosive material in LS-DYNA. The JWL equation of state used to describe the properties of explosives is as follows:

$$P_{0} = A\left( {1 - \frac{\omega }{{R_{1} V}}} \right){\text{e}}^{{{\text{ - R}}_{{1}} V}} + B\left( {1 - \frac{\omega }{{R_{2} V}}} \right){\text{e}}^{{{\text{ - R}}_{{2}} V}} + \frac{{\omega E_{0} }}{V}$$

where $P_{0}$—Detonation pressure, Pa.$A$, $B$, $R_{1}$, $R_{2}$—Explosive characteristic parameters. $V$—Relative transmitting response volume. $E_{0}$—Initialize internal energy, Pa.

According to a large number of open-pit mine blasting data, most of the mines currently use 2^#^ emulsion explosives. Therefore, the explosive parameters in the simulation model are set as shown in Table 5, and the parameters of the explosive state equation are shown in Table 6.

**Table 5:** Explosive material model parameters.

Name of explosive	Density (kg m^−3^)	Detonation velocity (m s^−1^)	Detonation pressure (GPa)
Emulsified explosive	1.1 × 10^3^	4500	5.6

**Table 6:** Characteristic parameters of explosives.

A (GPa)	B (GPa)	$R_{1}$	$R_{2}$	$\omega$	$E_{0}$ (GPa)
214	0.18	4.2	0.9	0.15	4.19

**Figure 4:**
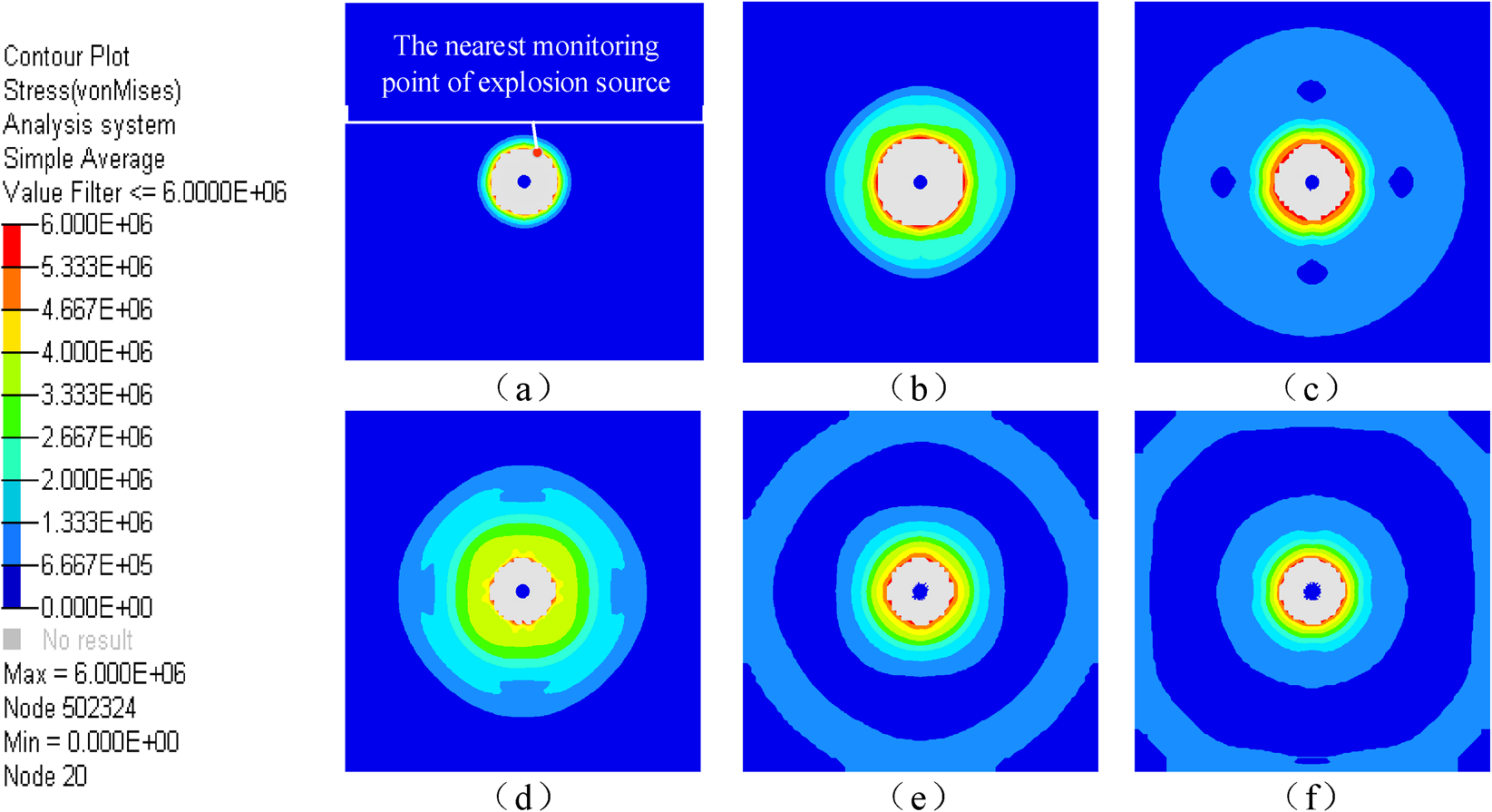
Simple view of blasting process.

Explosive explosion simulation process, as shown in Fig. 4.

**Figure 5:**
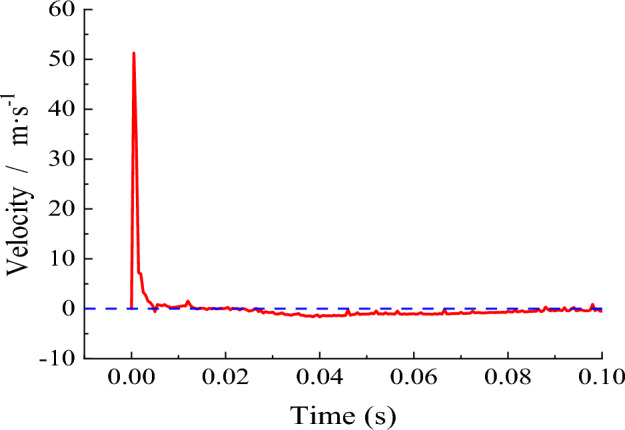
Time-history curve of blasting speed.

Through the LS-PROPOST post-processing software, the unit closest to the blast hole is selected as the monitoring unit, and the blasting speed time history curve (Fig. 5) is extracted. The data is converted into a file that can be identified by the PFC software and imported into the model sample established in the PFC, which is applied to the hole wall.

#### Explosive gas application

**Figure 6:**
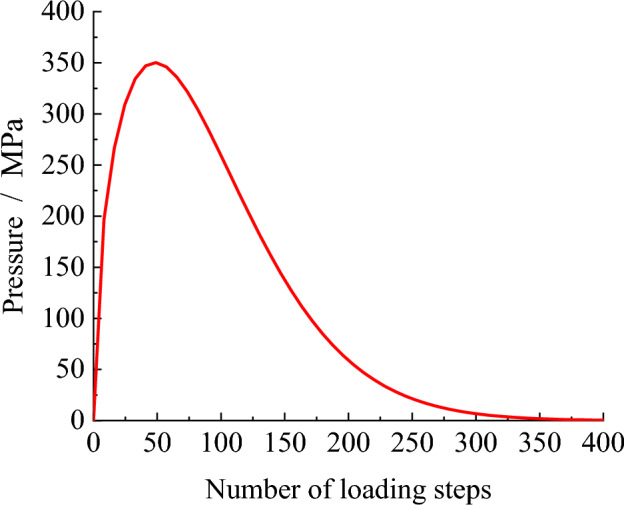
Pressure loading curve of explosive gas.

After adding the explosion stress wave in the FISH language of PFC, it is necessary to apply the detonation gas to the hole wall to realize the destructive effect of the detonation gas in the explosion process. The detonation gas pressure acting on the borehole wall is given according to the literature^21,22^. The load form is expressed by the Weibull distribution function, and the loading curve is shown in Fig. 6.

$$p_{g} = p_{0} \left( {{n \mathord{\left/ {\vphantom {n {n_{0} }}} \right. \kern-0pt} {n_{0} }}} \right)^{{m_{1} - 1}} \exp \left[ { - \left( {{n \mathord{\left/ {\vphantom {n {n_{0} }}} \right. \kern-0pt} {n_{0} }}} \right)^{{m_{1} }} } \right]$$

where $p_{g}$—Explosion gas pressure, Pa. $m_{1}$—Shape parameter, value as1.5. $n$—The number of loading steps of $p_{g}$_._ $p_{0}$, $n_{0}$—constant, value as $n_{0} = 100$, $p_{g0} = 47$ GPa。

### Formation and attenuation of explosion stress wave

#### Formation and attenuation of explosion stress wave

**Figure 7:**
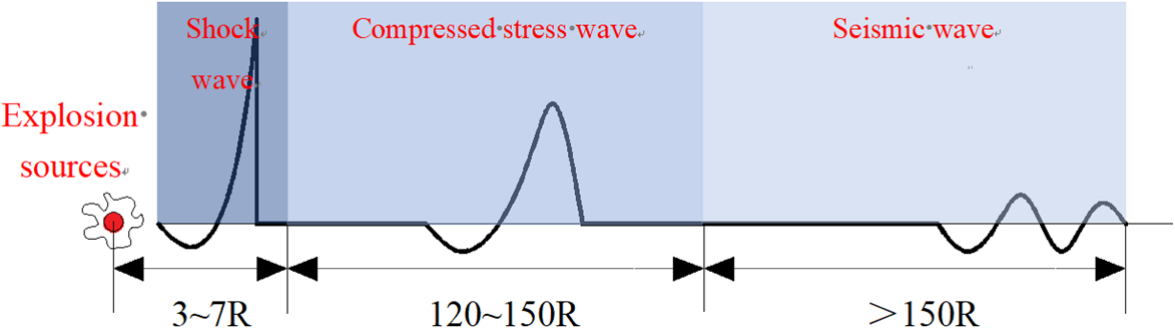
Evolution process of explosion stress wave.

The strong shock wave generated in the rock after the explosion of the explosive propagates in the distance at a supersonic speed, and the propagation distance is generally 3–7 R. As the shock wave propagates, its stress amplitude and wave velocity gradually decrease and gradually decay into compressive stress wave. The propagation distance of compressive stress wave is generally 120–150 R. When the strength of the compressive stress wave attenuates to the point where it can no longer damage the rock, it will be transformed into seismic waves. The formation and attenuation process is shown in Fig. 7 below.

The shock wave in rock will rapidly attenuate into stress wave. The radial stress attenuation law caused by any point in the rock mass within the scope of shock wave can be expressed by the following formula^23,24^.

$$\mathop \sigma \nolimits_{{\text{r}}} = {\text{P}} \overline{r}^{ - \alpha } ;\;\;\alpha = 2 \pm \frac{{\mu_{d} }}{{1 - \mu_{d} }}$$

where $\overline{r}$—scaled distance, Calculate the ratio of the distance from the point to the borehole to the radius of the borehole. $\mathop \sigma \nolimits_{r}$—radial stress, MPa. $\alpha$—Stress wave attenuation coefficient. ± —Shock wave zone/stress wave zone. $\mu_{d}$—Dynamic Poisson 's ratio of rock, $\mu_{d} { = }0.8\mu$.

#### Verification of stress wave attenuation law in numerical simulation model

**Figure 8:**
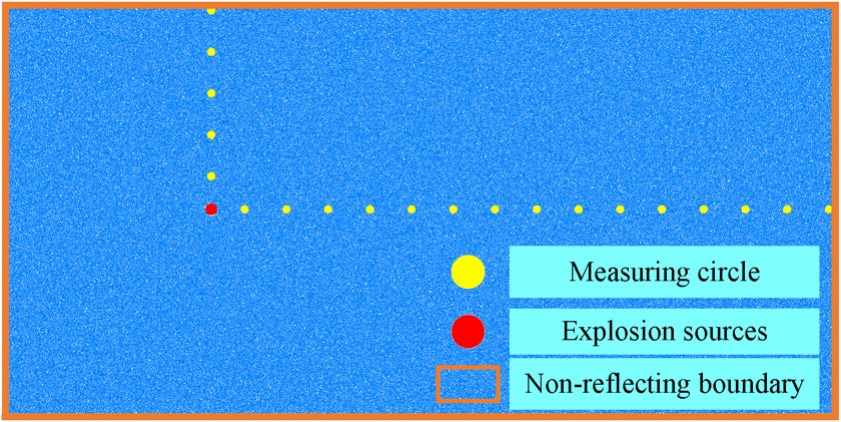
Simulation diagram of stress wave measurement.

As shown in Fig. 8 below, a numerical model with a size of 20 × 10 m is established to simulate the two-dimensional plane of the blasting area, and the particles near the upper, lower, left and right boundaries of the model are constrained.

The incident wave energy is absorbed by applying a load on the particles at the boundary to simulate the propagation of stress waves in an infinite medium. The explosion source is set at the midline of the upper and lower boundaries 5 m away from the left boundary, with a radius of 0.1 m and an initial pressure of 50 MPa. Fifteen measuring circles are set at an interval of 1 m on the middle line of the upper and lower boundaries on the right side of the explosion source to monitor the stress.

**Figure 9:**
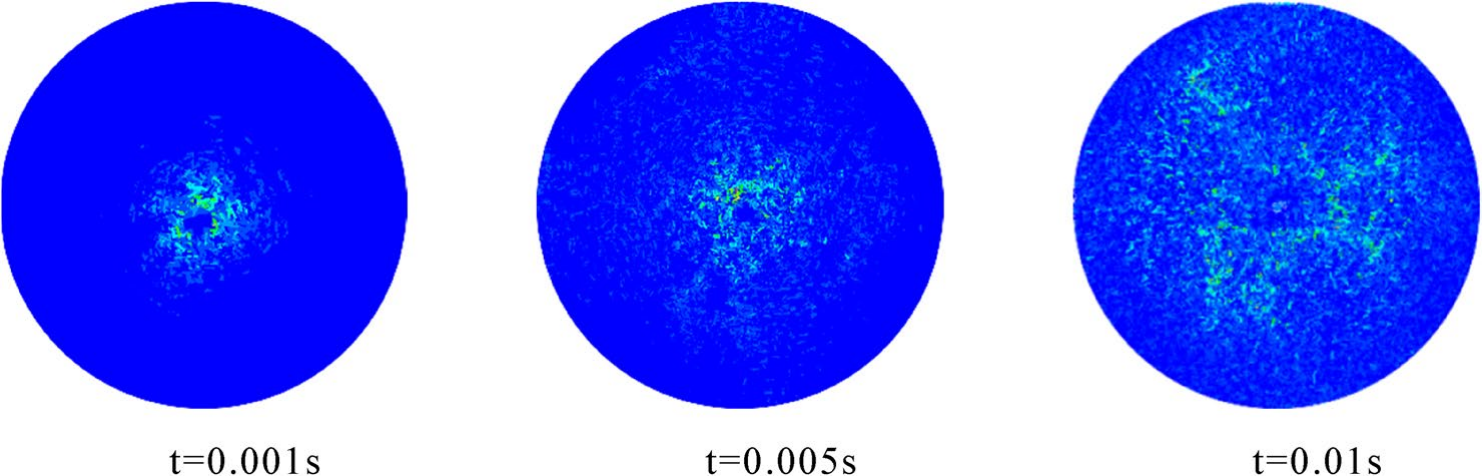
The propagation of stress wave during explosion in simulation.

The stress propagation process in the explosion process is shown in Fig. 9.It can be seen from the figure that the stress wave of the rock mass around the explosion source after the explosion is circularly symmetrically distributed, and it continues to propagate to the distance at a certain speed in the infinite medium, which is the same as the previous research results. The feasibility of blasting simulation using LS-DYNA and PFC is preliminarily verified.

**Figure 10:**
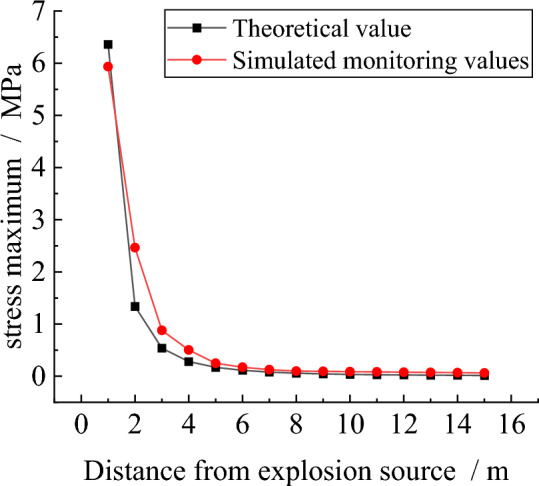
Theoretical—Simulation stress wave attenuation curve.

In order to further verify the reliability of the numerical simulation method used in this paper, the stress peaks monitored by each measurement circle in the model are statistically analyzed to obtain the attenuation curve of the stress wave in the simulation. In addition, according to the formula (stress wave attenuation formula), the calculated value of the stress wave is obtained, and the comparison curve shown in Fig. 10 is obtained. Through the comparison between the theoretical value and the simulated value, it can be seen that the attenuation law of the stress value of the two is roughly the same, which verifies the accuracy of the simulation method.

## Study on blasting mechanism of burnt rock

### Reflection and refraction of stress wave in jointed rock mass

According to the previous analysis, the structural planes (including faults, beddings, joints and fissures) in the rock mass play a role in stress concentration, enhancing stress wave reflection, absorbing explosive energy, venting energy, wedging and changing the direction of blasting during the blasting process, which seriously affects the blasting effect.The structural plane in the rock mass can be regarded as a filling body with relatively poor physical and mechanical parameters compared with the conventional rock mass. Some are consolidated rocks with slightly higher strength, and some are loose rock and soil bodies whose strength is not urgent.The existence of structural plane leads to the anisotropy of rock. Based on the various effects of structural plane in the blasting process, it hinders and attenuates the propagation of stress wave and energy, and then affects the final effect of rock blasting.

According to the different incident angles when the stress wave propagates to the structural plane, the reflection and refraction of the stress wave in the structural plane are different in the two cases of vertical incidence and oblique incidence. However, regardless of the incident mode, the existence of the structural plane directly affects the propagation velocity of the wave in the rock mass. The smaller the angle of the stress wave incident on the structure, the more it affects the propagation of the stress wave. The reflection and refraction of the stress wave in the structural plane reduces the amplitude of the stress wave, and the attenuation rate increases with the increase of the number of structural planes and the stiffness of the structural plane.

### Random fracture network model parameters of burnt rock mass

In PFC, the fracture network is constrained accordingly. In the two-dimensional case, the dip angle is a clockwise rotation angle in the positive direction of the x-axis. The microscopic characteristics, location and arrangement of cracks in rock mass are random numbers. The mathematical description of different distributions in Weibull random distribution model is as follows:

$$x_{i} = \beta \left[ {\ln \left( {1 - R_{i} } \right)} \right]^{{{1 \mathord{\left/ {\vphantom {1 \alpha }} \right. \kern-0pt} \alpha }}}$$

Through the self-compiled FISH language, a random crack obeying the weibull distribution is inserted into the PFC, and a burnt rock model with multiple joint cracks and irregular distribution is constructed.

Under the long-term action of thermal stress and ground stress, a large number of joint fissures are produced in the burnt rock mass, with different shapes and random distribution. Compared with the general fractured rock mass, it is difficult to obtain the distribution of joint fissures inside the burnt rock through geological survey.In the field investigation of the internal fracture development of burnt rock mass in the fire area, the author tried to use the window method to carry out the fracture statistics. The results showed that the burnt rock mass structure was loose, the cementation was strong, and the difference between the rock mass and the rhyolite was huge, and the fracture statistics could not be realized.In addition, it is well known that the fractures in rock mass are the products of many complex geological processes in the geological history, which have the characteristics of the diversity of influencing and controlling factors, the randomness of development and distribution. Therefore, in this numerical simulation, it is considered that the cracks in the burnt rock mass are randomly distributed, and the discrete crack (DFN) model used in this numerical simulation is currently recognized by many scholars as a model suitable for characterizing rock mass cracks. In this model, the distribution pattern, crack trace length, and crack density of joint cracks can be specified^25^.

#### Joint fissure occurrence

The occurrence of joint fissures is the distribution form, which is a description of the spatial orientation state of the fissure surface in the burnt rock. Weibull distribution is the most widely used fracture distribution form at present. Therefore, in this numerical simulation, the straight line segment is used to represent the joint fissures in the burnt rock, and the diagonal line in the model is taken as the distribution center, and the random fissures obeying the Weibull distribution are inserted.

#### Joint crack trace length

According to the on-site exploration and consultation of the staff responsible for blasting in the mining area, it is known that there are few penetrating cracks in the burnt rock mass, most of which are inclusive or intersecting joint cracks. Moreover, due to the serious cementation in the burnt rock mass, the internal cracks are mostly closed cracks. Therefore, in this numerical simulation test, in order to simulate the actual situation of cracks in the burnt rock mass, the length of cracks in the rock mass is set to be randomly generated in the range of 0.4–2 m.

#### Joint fissure density

In the discrete fracture network, the density of the two-dimensional model fracture generally uses the professional term P10 to represent the density of the joint fracture, that is, the number of cutting fractures per unit length. Dershowitz and Herda^26^ classified the fracture density measurement according to the measurement area dimension and the fracture dimension. The density measurement method of P10 in the two-dimensional model is based on the number of cracks in the unit length borehole.

**Figure 11:**
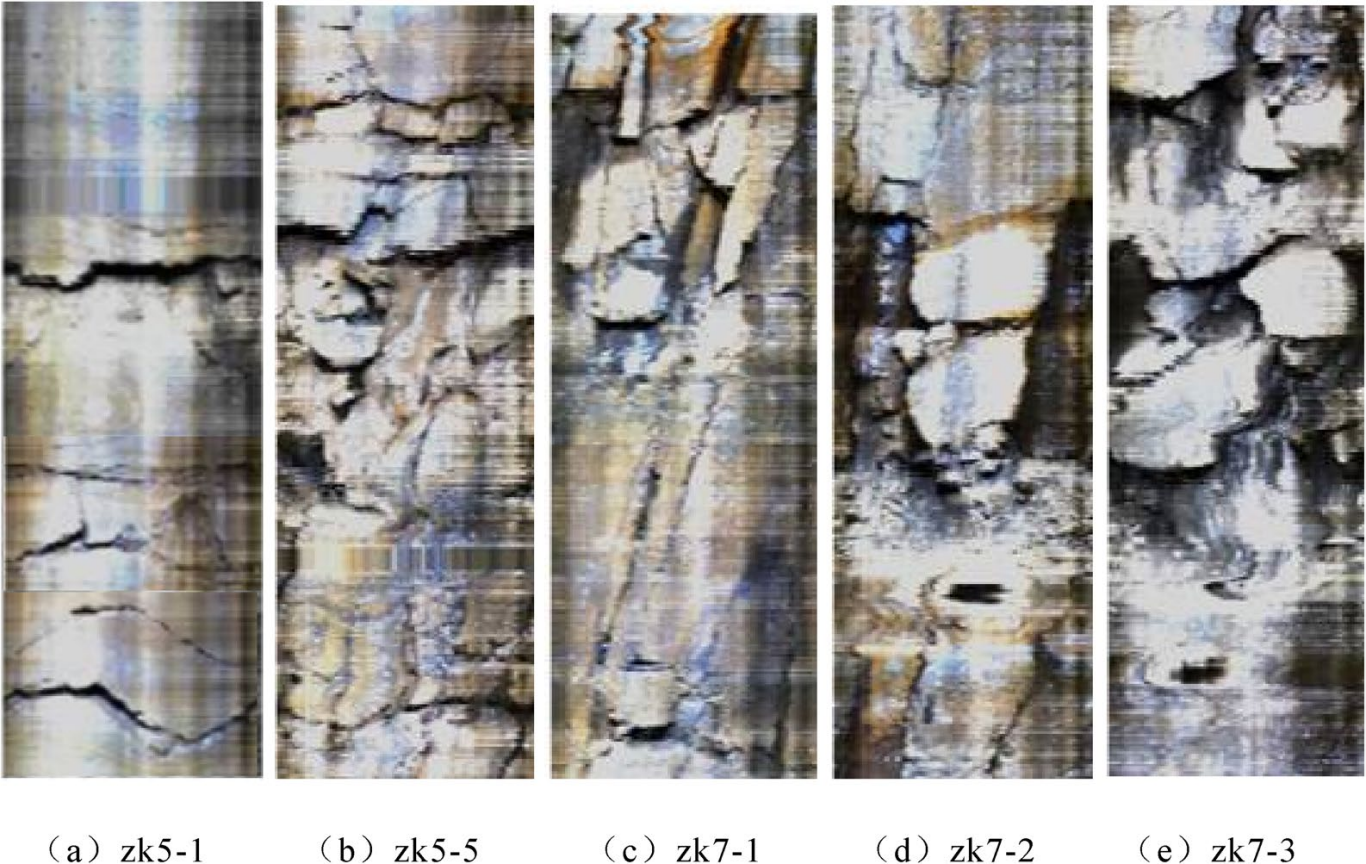
Distribution of cracks in boreholes.

Because the core taken out of the fire area is extremely broken, the recovery rate is generally low. Therefore, the fracture density in this simulation is based on the monitoring results of borehole peeping (As shown in Fig. 11), and the joint fracture density in the numerical model is set. According to statistics, the number of cracks in five 1 m-long boreholes is shown in Table 7 below. Because the burnt rock mass is loose and there is a certain cementation ability inside, only the obvious or interlayer cracks are considered when counting the number of cracks in the borehole.

**Table 7:** Number of cracks in burnt rock drilling core.

Drilling number	zk5-1	zk5-5	zk7-1	zk7-2	zk7-3
Fracture number	7	9	6	8	8

According to the number of cracks in each drill core in the table, it can be seen that the value of P10 should be between 0.6 and 0.9.

For the inserted random cracks, certain parameters should also be given, as shown in Table 8.

**Table 8:** Microscopic parameters of fractures.

Mesoscopic parameters	kratio	pb_kn	pb_kratio	pb_fa	pb_coh	pb_ten
Values	1.3	5e12	1.3	0.70	0.0	0.0

Because the existence of joint cracks in burnt rock has a serious impact on the blasting effect, the blasting results should be different under different crack densities. Therefore, different P10 values are used to establish the numerical model. Firstly, the influence of different fracture density on the blasting effect is analyzed, so as to determine the fracture density adopted by the numerical model for the subsequent analysis of the blasting fracture mechanism of burnt rock.

**Figure 12:**
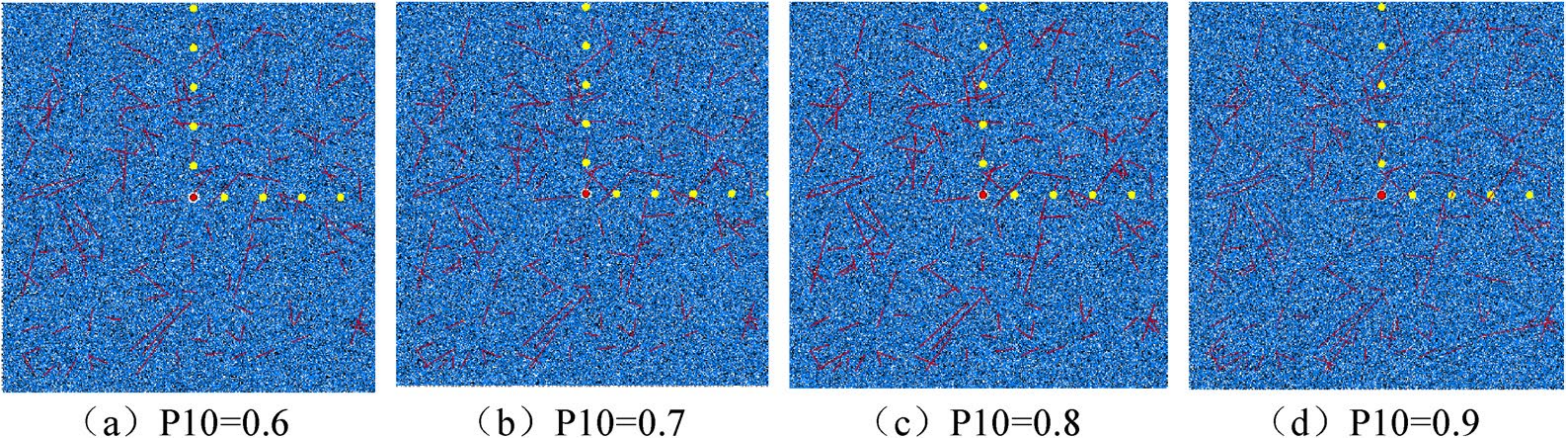
Numerical model under different fracture densities.

Using the numerical simulation method described in the previous article, a 10 m × 10 m model is first established, and then the microscopic parameters are set by parameter calibration, the blasting load is applied, the viscous boundary and damping are set, and the random cracks such as the above corresponding parameters are inserted. The numerical model under different densities shown in Fig. 12.

**Figure 13:**
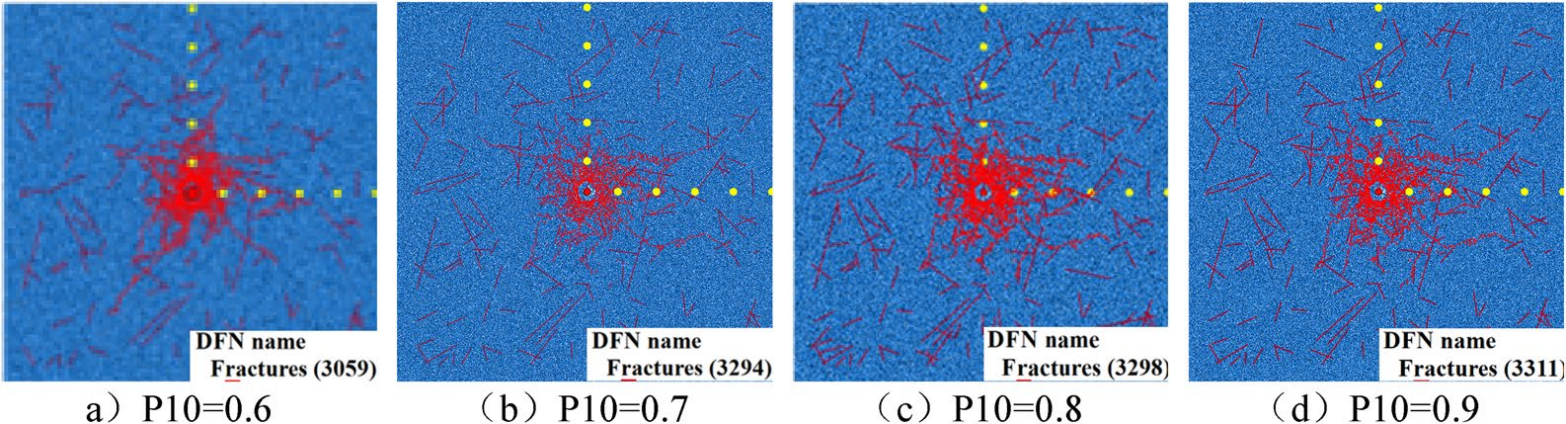
Explosion results of numerical model under different fracture density.

In the model, the red straight line segment represents the joint fissure, the red origin is the explosion center point, and the yellow origin is the stress monitoring point. A viscous boundary is set around the model to simulate the propagation of explosives in an infinite medium, and the surrounding boundary does not reflect the stress wave. The explosion results of numerical models with different fracture densities are shown in Fig. 13.

Figure 13 Explosion results of numerical model under different fracture densities.

From the explosion results under different crack densities in the above figure, it can be seen that as the crack density increases, the number of blast-induced cracks gradually increases, but the number of cracks is basically the same in the case of P10 = 0.7 and P10 = 0.8, while the crack growth rate at P10 = 0.9 does not increase significantly. From the perspective of the range of the fracture zone, due to the different random distribution of the joint cracks, the crack propagation under the same stress is also different. The extension length of the radial crack is related to the angle of the crack.In summary, combined with the actual situation of burnt rock, it is considered that the joint fracture density P10 should be 0.8 when the numerical model of burnt rock is established.

### The influence of random fractures in burnt rock

**Figure 14:**
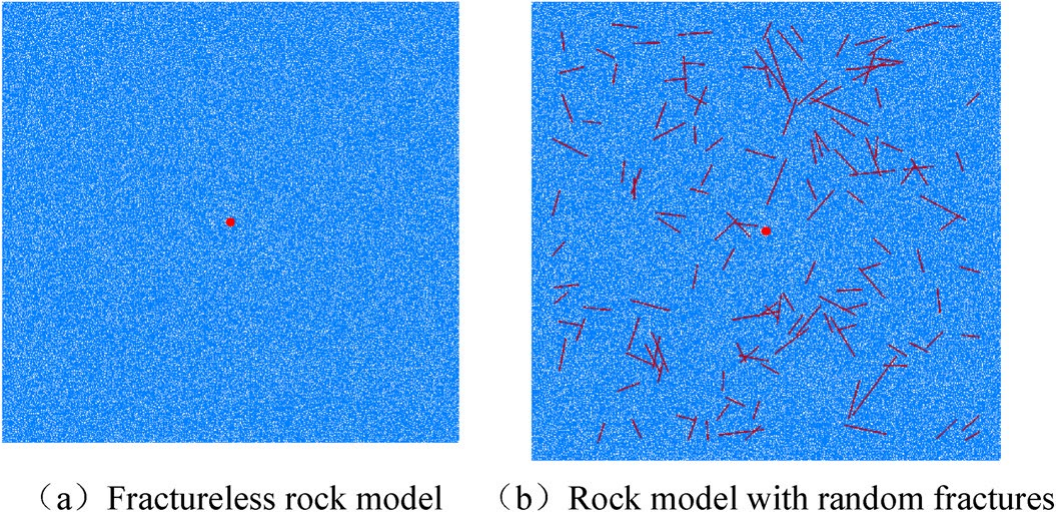
Establishment of rock model in simulation.

In this section, in order to analyze the influence of random cracks in burnt rock on blasting effect, a crack-free model and a model with random cracks based on the mechanical parameters of burnt rock are established. The model size is still 10 m × 10 m, the explosive diameter is 200 mm, the initial pressure of explosive is 100 MPa, and the origin of the yellow point is the stress monitoring point, as shown in Fig. 14 below. The crack propagation and stress distribution of the two models after explosion are compared. Combined with the theory of stress wave propagation, the influence of joint cracks on the internal action of explosives is comprehensively analyzed.

1. Crack propagation

**Figure 15:**
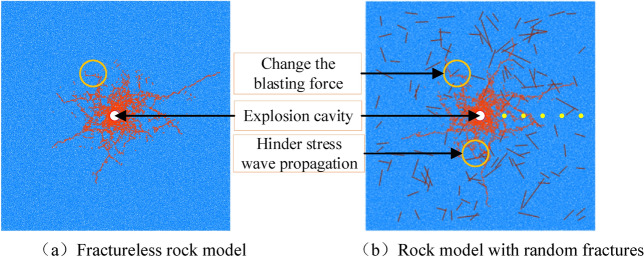
Crack propagation after explosion.

The simulation results are 0.1 s after the explosion of the explosive. At this time, the explosion process of the explosive is already over. The final crack propagation is shown in Fig. 15 below.

The damage inside the rock after the explosion of the explosive can be clearly shown in Figure (a), and the crushing area and the fracture area are obvious. Because the viscous boundary is set in the model, there is no reflection of the stress wave. Under the combined action of explosion stress wave and detonation gas, the explosion cavity is first formed, and then the crushing area is formed under the action of compression stress wave, and the effect of detonation gas in this area is not obvious. After the shock wave rapidly decays into a stress wave, the stress wave at this time cannot meet the compression failure conditions of the rock. It can only destroy the rock by causing the rock to move, forming radial tensile stress and circumferential tensile stress. With the propagation of the detonation gas, the gas wedge extrusion of the detonation gas further extends and expands the generated radial cracks, thus forming the explosion result shown in (a) above.

Because there are many joint cracks in burnt rock and they are random, the rock model is established by inserting random cracks in the simulation to simulate the blasting fragmentation of burnt rock, as shown in Figure (b). It can be seen from the diagram that in the burnt rock model, the radial crack generated by the explosion is different from the radial crack propagation direction in the non-random model due to the stress concentration of the stress wave, the enhancement of the stress wave reflection, and the change of the blasting direction. The angle and length of the extension are changed. If the crack angle is consistent with the stress wave propagation direction, the radial crack will continue to propagate with the crack direction. When the crack angle and the stress wave propagation direction have a certain angle, the refraction and reflection of the stress wave will hinder the propagation of the stress wave.

Due to the reflection enhancement effect of random cracks, the stress wave is reflected and refracted when it first encounters the crack, and it repeatedly acts between the blast hole and the crack, resulting in a higher degree of fragmentation of the compression failure area around the blast hole, a greater energy consumption of the shock wave, and a shorter stress wave. The action time, under the action of the same amount of blasting, the damage range is smaller, and the phenomenon of ' internal fragmentation and external fragmentation ' of the rock fragmentation is generated.

(2) Distribution of stress

**Figure 16:**
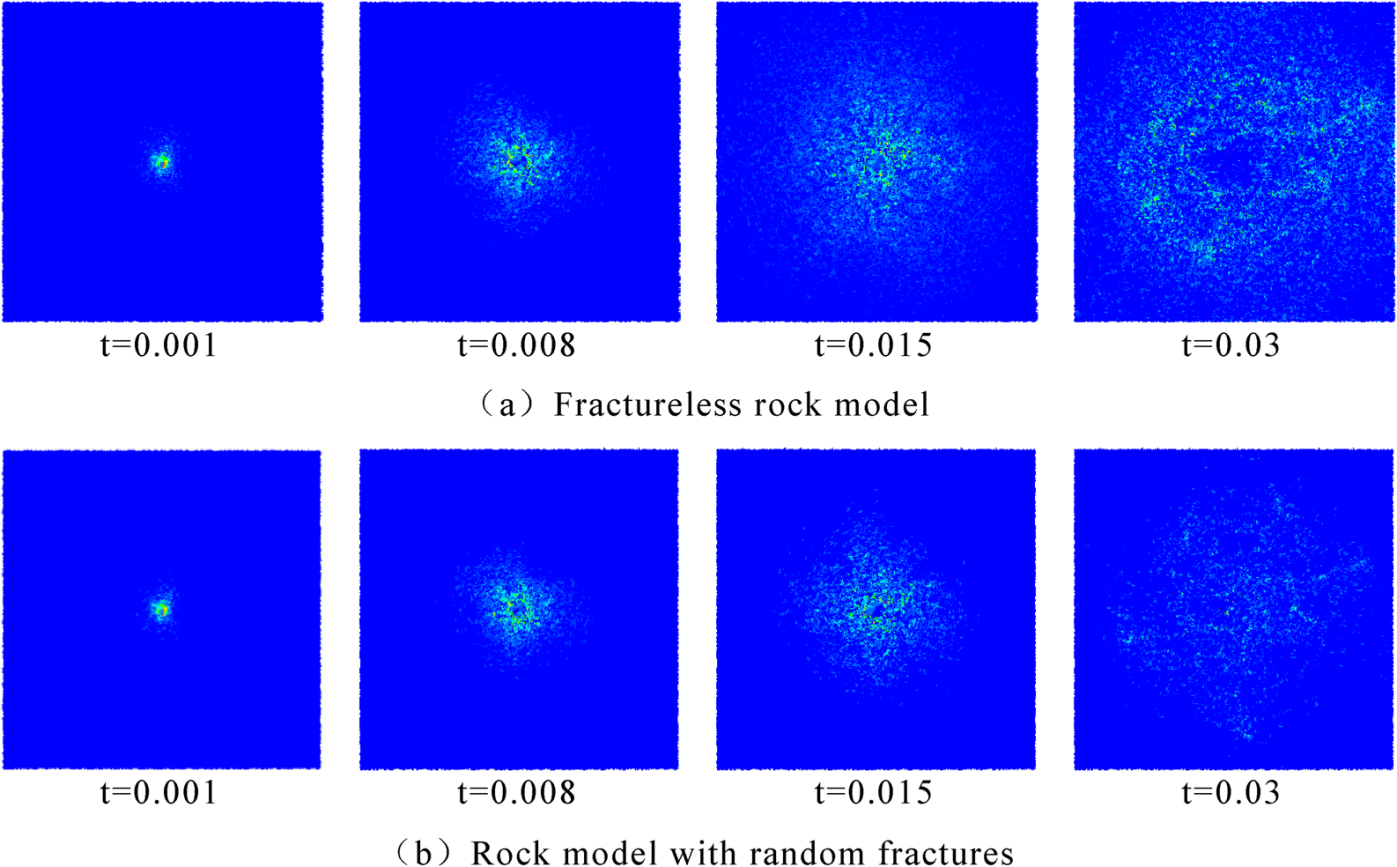
Propagation of stress wave in rock.

Figure 16 shows the stress propagation in the two models at the same time. It can be seen from the diagram that the stress propagation range is wider and more uniform without random cracks, while the propagation of stress wave is hindered by random cracks in burnt rock, and the stress intensity is reduced due to the energy absorption of joint cracks, which affects the propagation range of stress wave.

**Figure 17:**
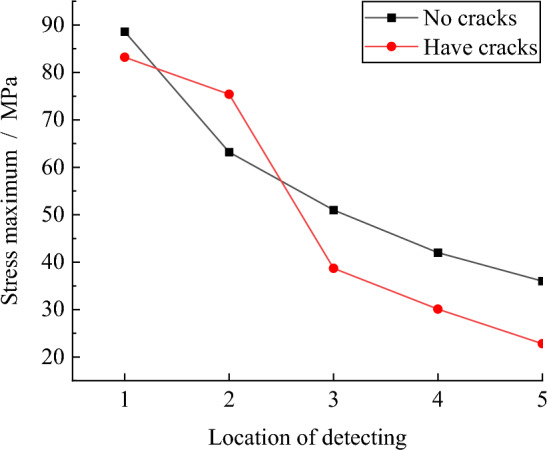
Peak stress monitoring.

Figure 17 below is the peak stress at the monitoring point. At the beginning of the explosion, because the stress wave did not propagate to the joint fissure, the peak stress at the monitoring point 1 was approximately the same, both of which were about 85 MPa; at the second monitoring point, due to the existence of joint fissures, the stress wave is reflected and superimposed, resulting in the stress peak at the second monitoring point of burnt rock is greater than the stress peak of conventional rock, which also shows that the burnt rock is more damaged in this area, which corresponds to the crack propagation results. With the decrease of shock wave and the effect of joint fissure, the peak stress in burnt rock decreases faster and lower in the subsequent propagation process.

### Mechanism of bench deep hole blasting in burnt metamorphic rock

Compared with the conventional rock step, the burnt rock step contains a large number of joint cracks, which seriously affects the propagation of stress wave and crack propagation. In addition, due to the reflection and superposition of joint cracks on stress wave, the damage degree of rock near the borehole is aggravated.

**Figure 18:**
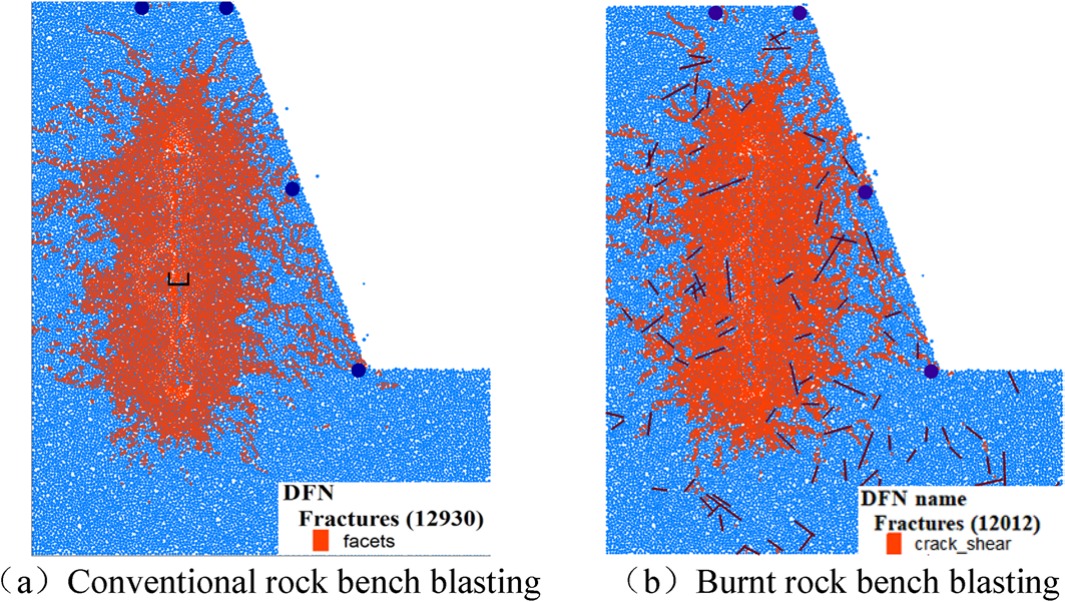
Simulation results of deep hole blasting.

According to the characteristics of fracture development in burnt rock, by using the numerical simulation method described above, the FISH language is compiled to simulate the blasting process of conventional rock bench and burnt rock bench respectively. The explosion results are shown in Fig. 18 below.

In the above figure, orange is an explosion crack, dark blue circle is a stress monitoring point, the width of the top surface of the step slope is 5.5 m, the depth of the blast hole is 10.5 m, and the height of the charge is 6.5 m. A sticky boundary is set on the left boundary of the model to simulate the infinite medium boundary. From the results of Fig. 18a, it can be seen that the crushing zone is about 7R (hole diameter), which is basically consistent with the theory of deep hole bench blasting. In the early stage of the explosion, under the combined action of stress wave and explosive gas, the surrounding rock is compressed and destroyed, and the explosion cavity is generated around the blast hole. With the propagation of energy, the rock steps are destroyed, and the explosion results are formed. A total of 12,930 cracks were formed, but limited to the current technical means, the annular cracks could not be clearly shown.

Figure 18b is based on Fig. 18a, other conditions remain unchanged, and random cracks (purple cracks in the figure) are inserted into the model to simulate the burnt rock steps. It can be seen from the blasting results in the figure that a total of 12,012 cracks (including inserted prefabricated random cracks) were formed in the burnt rock steps. The addition of random cracks affected the expansion of cracks in the crack zone, and produced stress concentration at the prefabricated cracks, resulting in cracks continuing to expand along the crack direction. Due to the different angles of the model cracks, the obstacles in the cracks near the vertical and borehole directions are more obvious. In addition, due to the reflection of cracks on stress waves, the compression zone of burnt rock steps is slightly larger than that of conventional rock steps. In summary, due to the influence of random joint fissures in burnt rock steps, the extension distance of explosive cracks is reduced, and only a few cracks are extended in a position where the direction of stress wave propagation is consistent with the direction of stress wave propagation.

**Figure 19:**
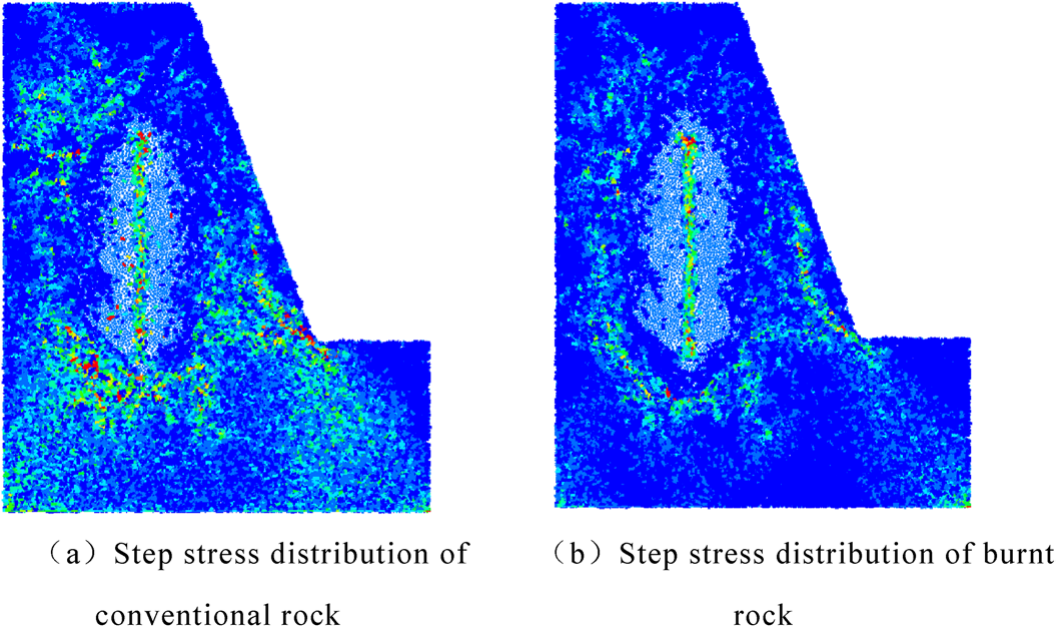
Comparison chart of stress distribution.

The stress distribution of the two models at the same time is intercepted, and the stress distribution is shown in Fig. 19. According to the comparison of stress distribution, it can be seen that under the same blasting time, the stress distribution range in the conventional rock step is larger and the strength is higher, while the burnt rock step has a slightly smaller propagation range and uneven stress distribution due to the influence of joint cracks. From the range of particles without stress around the hole in the picture, at the same time, the rock damage area around the burnt rock step hole is larger, the degree of crushing is higher, and the energy consumption of explosives increases in this area.

**Figure 20:**
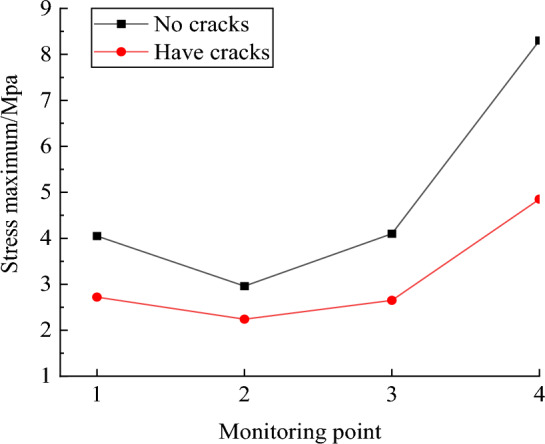
Peak stress comparison results.

The peak stress at each monitoring position can be obtained by measuring the circle in the model. The peak stress in the Y direction at each monitoring point is shown in Fig. 20. It can be seen from the figure that the peak stress of each monitoring point in the conventional rock step is greater than that of the burnt rock step. This is due to the fact that the stress wave is hindered by the joint cracks in the rock during the propagation process, which reduces the action time of the stress wave in the rock step and absorbs a certain amount of energy. If the same blasting design conditions are adopted, the failure conditions of the rock at the free surface cannot be fully satisfied, resulting in large blocks, 'hard ribs', 'umbrella eaves', etc.

## Conclusion

The existence of burnt rock causes the bench deep hole blasting operation in the open-pit mine fire area to be affected by joints and fissures, and the blasting effect is not ideal, which is an urgent problem to be solved in the current open-pit mine burnt rock blasting. In this paper, burnt rock is taken as the research object. Based on the field investigation and data collection of open-pit mines with fire areas, the blasting and crushing mechanism of burnt rock is deeply studied by means of theoretical analysis, laboratory experiment, numerical simulation and numerical calculation. The main conclusions are as follows:

1. The test results of physical and mechanical parameters and mineral composition of burnt rock show that with the increase of the distance between the rock layer and the spontaneous combustion coal seam, the change of mineral composition in the rock is smaller, the change of rock strength is obvious, the rock is brittle and fragile, and the cracks in the rock mass are very developed. Because the rock mass is mainly subjected to tensile failure during the blasting process (the tensile strength of burnt rock is high), and is seriously affected by joint fissures, the difficulty coefficient of blasting fragmentation of burnt rock mass increases.
2. The LS-DYNA/PFC2D joint numerical simulation method is used to simulate the blasting failure process of burnt rock under the combined action of stress wave and detonation gas. The results show that when the direction of joint fracture is parallel to the direction of stress wave propagation, the stress concentration phenomenon leads to the first crack at the crack tip, and most of the detonation gas is wedged in until it is consumed or washed out of the rock mass. When the angle is vertical or inclined, the reflection and refraction of the stress wave at the joint fracture shortens the action time of the stress wave, aggravates the crushing degree of the rock around the borehole, and consumes a large amount of detonation gas energy.
3. The addition of random cracks affects the propagation of blast-induced cracks in the crack area, and produces stress concentration, which leads to the continuous propagation of cracks along the crack direction. Due to the different crack angles of the model, the cracks in the direction close to the vertical and borehole directions play a more obvious hindering role. In addition, due to the reflection of cracks on stress waves, the compression zone of burnt rock steps is slightly larger than that of conventional rock steps.
4. Because the stress wave is hindered by the joints and fissures in the rock during the propagation process, the action time of the stress wave in the rock step is reduced, and a certain amount of energy is absorbed, resulting in a lower stress peak at the edge of the burnt rock step. In the case of the same charging method, structure and dosage, the burnt rock step cannot be completely destroyed.

## Data Availability

The raw data supporting the conclusion of this article will be made available by the authors, without undue reservation. Zheng-Zhao Jia (Jiazz_1994@163.com) should be contacted if someone wants to request the data from this study.
